# Supplementary figures and images for: The Drug Susceptibility of Non-Tuberculous Mycobacteria (NTM) in a Referral Hospital in Rome from 2018 to 2023
Source: Microorganisms. 2024 Aug 8;12(8):1615. doi: 10.3390/microorganisms12081615 (PMC11356625; doi:10.3390/microorganisms12081615)

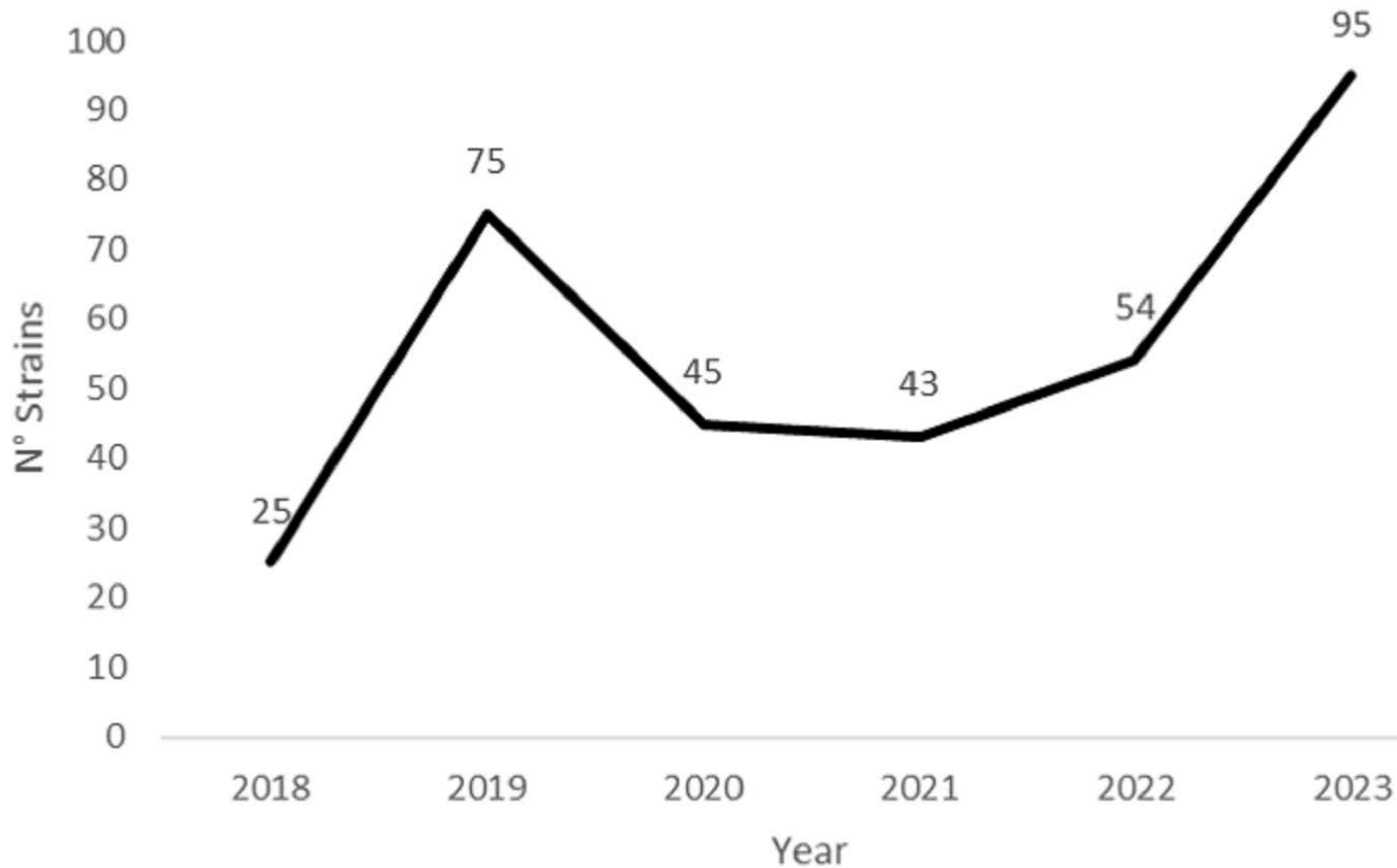

Supplement: Supplementary file 1 [file microorganisms-12-01615-s001.zip › Figure S1.pdf]
